# Definition of Mesh Weight and Pore Size in Groin Hernia Repair: A Systematic Scoping Review of Randomised Controlled Trials

**DOI:** 10.3389/jaws.2023.11179

**Published:** 2023-04-13

**Authors:** Can Deniz Deveci, Stina Öberg, Jacob Rosenberg

**Affiliations:** Centre for Perioperative Optimisation, Department of Surgery, Herlev Hospital, University of Copenhagen, Copenhagen, Denmark

**Keywords:** inguinal hernia, groin hernia, femoral hernia, lightweight mesh, heavyweight mesh

## Abstract

**Introduction:** Groin hernia literature often uses the terms light- and heavyweight and small or large pores to describe meshes. There is no universal definition of these terms, and the aim of this scoping review was to assess how mesh weight and pore sizes are defined in the groin hernia literature.

**Methods:** In this systematic scoping review, we searched PubMed, Embase, and Cochrane CENTRAL. We included randomised controlled trials with adults undergoing groin hernia repair with the Lichtenstein or laparoscopic techniques using a flat permanent polypropylene or polyester mesh. Studies had to use the terms lightweight, mediumweight, or heavyweight to be included, and the outcome was to report how researchers defined these terms as well as pore sizes.

**Results:** We included 48 studies with unique populations. The weight of lightweight meshes ranged from 28 to 60 g/m^2^ with a median of 39 g/m^2^, and the pore size ranged from 1.0 to 4.0 mm with a median of 1.6 mm. The weight of heavyweight meshes ranged from 72 to 116 g/m^2^ with a median of 88 g/m^2^, and the pore size ranged from 0.08 to 1.8 mm with a median of 1.0 mm. Only one mediumweight mesh was used weighing 55 g/m^2^ with a pore size of 0.75 mm.

**Conclusion:** There seems to be a consensus that meshes weighing less than 60 g/m^2^ are defined as lightweight and meshes weighing more than 70 g/m^2^ are defined as heavyweight. The weight terms were used independently of pore sizes, which slightly overlapped between lightweight and heavyweight meshes.

## Introduction

The standard treatment for symptomatic groin hernia is mesh repair (1). The rationale for using a mesh is the lower risk of recurrence compared with non-mesh repair (1), and the long-term reoperation rate is reported to be around 5% for mesh repairs (2). Despite the concern that meshes might introduce groin pain, a systematic review has shown that there is no difference in the risk of chronic pain regardless of repairing inguinal hernias with or without mesh (3). Therefore, the recommended and most used techniques are the Lichtenstein repair and the laparoscopic transabdominal preperitoneal (TAPP) and total extraperitoneal (TEP) techniques (1).

Today, there are many different meshes on the market, but the most used is a permanent flat polypropylene mesh (1). The terms lightweight, mediumweight, and heavyweight together with large and small porous have been used for many years to describe a mesh. Generally, a lightweight mesh has large pore size with less weight, whereas a heavyweight mesh has small pore size with more weight (1). Interestingly, systematic reviews have shown a lower risk of chronic pain when using a lightweight mesh in Lichtenstein repair (4) and a lower risk of recurrence when using a heavyweight mesh in laparoscopic repair (5). However, there is no clear definition of what the definition of a lightweight and heavyweight mesh is (1).

Due to the lack of agreement on mesh weight definitions, this systematic scoping review aimed to map how researchers conducting randomised controlled trials (RCT) on patients with groin hernias have defined lightweight, mediumweight, and heavyweight meshes in terms of areal weight and pore sizes.

## Methods

This systematic scoping review was reported using the Preferred Reporting Items for Systematic reviews and Meta-Analysis extension for Scoping Review (PRISMA-ScR) guideline (6). The protocol was registered at Open Science Framework (OSF) before data extraction was initiated (7).

The eligibility criteria were studies including participants minimum 18 years old undergoing groin hernia repair with a mesh using the Lichtenstein, TAPP, or TEP techniques. The mesh had to be flat and made of permanent polypropylene or polyester, and simple flat meshes are the most commonly used mesh type (1). And the studies had to use the terms lightweight, mediumweight, or heavyweight when describing the mesh. The outcome of this systematic scoping review was to report researchers’ definitions of light-, medium-, and heavyweight meshes. To define the mesh weight, we focused on areal weight in g/m^2^, but other definitions of weight were also considered. Furthermore, pore sizes were also included in studies where the weight was defined. An additional outcome was to report how many studies had used light-, medium-, and heavyweight meshes when repairing with the Lichtenstein, TAPP, or TEP techniques. We excluded studies that used meshes of other shapes than simple flat, such as special firm borders, 3D shapes, and self-gripping or adhesive meshes. If studies only mentioned using a light-, medium-, or heavyweight mesh but without further specifying the weight, the manufacturer’s website was searched to retrieve these data. We excluded studies if they failed to mention the term “weight.” We also excluded studies if the areal weight was insufficiently described in the study and it could not be found on the website of the manufacturer, regardless of whether they had reported the pore size or not. Studies that included other repairs than inguinal- or femoral hernia repairs or other meshes than flat polypropylene or polyester meshes were included if the results were separately presented for the eligible patients. Finally, only published randomised controlled trials written in English were included.

A search strategy was first created in PubMed with the help of an information specialist. This search strategy was later converted to the databases Embase and Cochrane CENTRAL. All searches were conducted on 19 August 2022. We also performed a snowball search by studying the reference lists of the included studies (8), and studies that seemed relevant were full text screened according to the eligibility criteria. The search strategy in PubMed was: “(femoral OR inguinal OR groin OR lateral OR medial OR pantaloon OR indirect OR direct) AND (hernia OR hernia [MeSH Terms]) AND (“randomized control trial” [Title/Abstract] OR “controlled clinical trial” [Title/Abstract] OR “randomized” [Title/Abstract] OR “randomised” [Title/Abstract] OR “RCT” [Title/Abstract] OR “trial” [Title/Abstract]).” After conducting the searches, studies were imported to the reference software Mendeley^1^ where duplicates were removed. The studies were screened using the software Covidence^2^, which also removed further duplicates. Both the screening of titles and abstracts and of full text papers were done by two authors independently. If there were any disagreement, it was resolved by discussion within the author group. If needed, study authors were contacted by e-mail twice for data clarity.

Data were first extracted for five studies to a pilot Excel spreadsheet by the first author. The pilot sheet was discussed within the author group, and after agreement on the final spreadsheet, the first author extracted data uniformly for all studies. The extracted data were first author, year of publication, number of eligible patients, type of groin hernia repair, type of groin hernia (inguinal or femoral), whether the mesh was defined as light-, medium-, or heavyweight, and mesh details such as weight in g/m^2^, pore size, and mesh size. Categorical data were presented with numbers and percentages, and continuous data were reported as median and interquartile range (IQR) and range. Pore sizes reported in mm^2^ were calculated to diameter in mm based on the formula to calculate the area of a circle and isolation of radius; “A = π·r^2^”.

## Results

**FIGURE 1:**
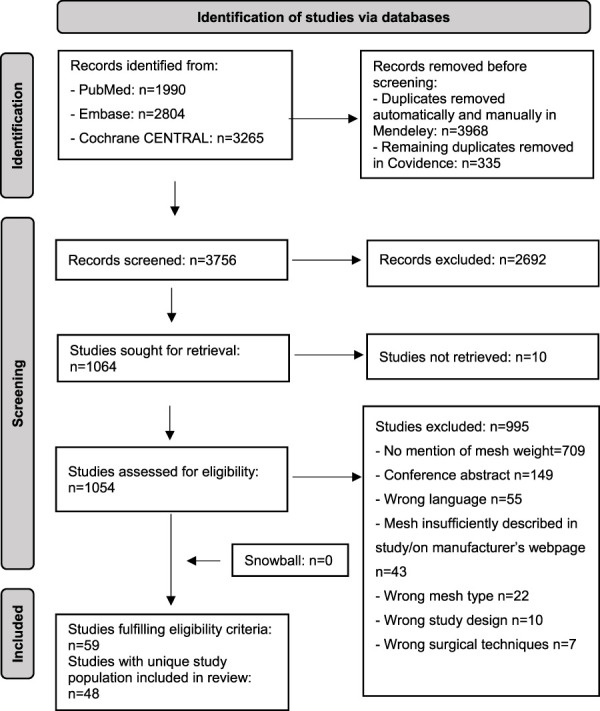
Preferred Reporting Items for Systematic reviews and Meta-Analysis extension for Scoping Review (PRISMA) flow diagram. n: number.

Study selection is illustrated in the PRISMA flowchart (Figure 1). We identified 8,059 records, and 1,054 of these were full text screened. Finally, 59 studies fulfilled the eligibility criteria (9–67). Of these studies, 11 had reused the patient population (57–67), which resulted in 48 studies with a unique population (9–56). Thus, only data from these 48 studies are presented in the following.

Study characteristics are presented in Table 1. The 48 randomised controlled trials (9–67) were published between 2003 and 2021. Thirty-seven studies used the Lichtenstein repair (10–29, 31, 32, 34–39, 43–46, 50–53, 55) and 12 studies used laparoscopic repairs (9, 28, 30, 33, 40–42, 48–49, 54, 56). Of these 12 studies, six used TEP repair (28, 33, 40–42, 54), four used TAPP repair (30, 48, 49, 56), and two studies used TEP and TAPP repairs (10, 47). Two of the 48 studies included patients with groin hernias (17, 41), and the remaining studies only included inguinal hernias. One study used a polyester mesh (13) while the remaining 47 studies used meshes made of polypropylene. Nine of the studies used two meshes (48–56), resulting in 48 studies mentioning mesh weight for 57 meshes. Of these 58 meshes, 26 were by the authors defined as lightweight meshes (9–28, 50–55), 30 as heavyweight meshes (29–56), and one as a mediumweight mesh (56). Even though three studies did not use the term heavyweight, we interpreted it as heavyweight since two studies described the mesh as conventional densely woven (46, 50) and one as non-lightweight with a high areal weight (42). The one study that defined their mesh as mediumweight had a weight of 55 g/m^2^ and a pore size of 0.75 mm (56).

**TABLE 1:** Study characteristics and mesh properties.

Ref	Study characteristics				Mesh characteristics				Brand
	Year	Patients^a^	Hernia	Repair type	Mesh	Weight (g/m2)	Pore (mm)	Size (cm)	
(9)	2016	140	Inguinal	TEP/TAPP	LW	30–45	> 2	10 x 15	Prolene soft
(10)	2021	20	Inguinal	Lichtenstein	LW	44	NR	NR	Prolene
(11)	2017	70	Inguinal	Lichtenstein	LW	36	1	NR	Optilene LP
(12)	2017	170	Inguinal	Lichtenstein	LW	46	NR	NR	Parietex
(13)	2017	370	Inguinal	Lichtenstein	LW	60	NR	7.5 x 15	Optilene
(14)	2016	63	Inguinal	Lichtenstein	LW	36	3.0–4	4.5 x 10	Optilene LP
(15)	2016	258	Inguinal	Lichtenstein	LW	38	NR	NR	Parietene Light
(16)	2016	151	Groin	Lichtenstein	LW	53.7	NR	NR	Parietene Light
(17)	2015	216	Inguinal	Lichtenstein	LW	60	NR	9 x 13	Optilene
(18)	2015	75	Inguinal	Lichtenstein	LW	36	1	6 x 14	Optilene LP
(19)	2014	287	Inguinal	Lichtenstein	LW	38	NR	NR	Parietene Light
(20)	2014	70	Inguinal	Lichtenstein	LW	36	1	4.5 x 10	Optilene LP
(21)	2013	159	Inguinal	Lichtenstein	LW	NR	NR	6 x 13.7	Soft mesh, Bard
(22)	2013	80	Inguinal	Lichtenstein	LW	48	NR	NR	Evolution P3EM
(23)	2012	196	Inguinal	Lichtenstein	LW	35	1.6	10 x 15	Prolene
(24)	2012	153	Inguinal	Lichtenstein	LW	<40	NR	NR	Parietene Light
(25)	2012	110	Inguinal	Lichtenstein	LW	52	NR	7.5 x 15	ProLite-Ultra
(26)	2011	302	Inguinal	Lichtenstein	LW	60	NR	9 x 13	Optilene
(27)	2011	110	Inguinal	Lichtenstein	LW	38	NR	NR	Surgimesh WN
(28)	2020	176	Inguinal	Lichtenstein/TEP	LW	38	1.6	10 x 15	Parietene Light
(29)	2020	43	Inguinal	Lichtenstein	HW	80–85	NR	6 x 12	Prolene
(30)	2020	54	Inguinal	TAPP	HW	>75	NR	10 x 15	NR
(31)	2018	197	Inguinal	Lichtenstein	HW	90	NR	NR	Bard Flatmesh
(32)	2017	25	Inguinal	Lichtenstein	HW	100	NR	NR	Marlex
(33)	2015	454	Inguinal	TEP	HW	80	0.8–1.2	10 x 15	Prolene
(34)	2014	113	Inguinal	Lichtenstein	HW	82	0.8	8 x 12	Prolene
(35)	2014	25	Inguinal	Lichtenstein	HW	85	NR	10 x 15	Prolene
(36)	2013	76	Inguinal	Lichtenstein	HW	100	1	NR	NR
(37)	2012	300	Inguinal	Lichtenstein	HW	>80	NR	NR	Prolene
(38)	2011	34	Inguinal	Lichtenstein	HW	100	1	8 x 15	NR
(39)	2011	16	Inguinal	Lichtenstein	HW	105	0.82	NR	Prolene
(40)	2010	20	Inguinal	TEP	HW	95	1	13 x 15	Marlex
(41)	2010	211	Groin	TEP	HW	105	0.8-1	12 x 15	Prolene
(42)	2010	40	Inguinal	TEP	HW	80	NR	10 x 15	Hi-Trex
(43)	2008	161	Inguinal	Lichtenstein	HW	>80	NR	10 x 15	Prolene
(44)	2006	301	Inguinal	Lichtenstein	HW	>80	NR	7.5 x 15	Prolene
(45)	2005	159	Inguinal	Lichtenstein	HW	85	1	NR	Prolene
(46)	2004	48	Inguinal	Lichtenstein	HW	100–110	NR	8 x 13	Atrium
(47)	2013	149	Inguinal	TEP/TAPP	HW	80–85	NR	10 x 15	Prolene
(48)	2008	120	Inguinal	TAPP	HW	108	1.0–1.6	10 x 15	Prolene
					HW	116	0.08–0.1	10 x 15	Serapen
(49)	2003	40	Inguinal	TAPP	HW	108	1.0–1.6	NR	Prolene
					HW	116	0.8–1.0		Serapen
(50)	2007	153	Inguinal	Lichtenstein	LW	55	NR	NR	Premilene Mesh LP
					HW	82			Premilene
(51)	2017	58	Inguinal	Lichtenstein	LW	43.7	2.8	7 x 15	Bard Davol
					HW	105.4	0.84	7 x 15	Bard Davol
(52)	2013	110	Inguinal	Lichtenstein	LW	36	2.6	7.5 x 15	Dynamesh
					HW	72	1.8	7.5 x 15	Dynamesh
(53)	2010	135	Inguinal	Lichtenstein	LW	36	1	4.5 x 10	Optilene
					HW	82	0.8	4.5 x 10	Premilene
(54)	2009	50	Inguinal	TEP	LW	<50	>1	12 x 15	NR
					HW	≈100	<1	12 x 15	NR
(55)	2009	25	Inguinal	Lichtenstein	LW	43	NR	NR	Surgimesh WN
					HW	80			Surgipro
(56)	2011	300	Inguinal	TAPP	MW	55	0.75	10 x 15	Premilene LP
					HW	90	1.2	10 x 15	Prolene

^a^ Only the numbers of eligible patients are presented; ref, reference; NR, not reported; TEP, total extraperitoneal; TAPP, transabdominal preperitoneal; LW, lightweight; HW, heavyweight; MW, mediumweight.

In the 37 studies where the Lichtenstein technique was used, 30 lightweight and 18 heavyweight meshes were used (Table 2). In 12 studies where laparoscopic techniques were used, 3 lightweight, 12 heavyweight, and 1 mediumweight mesh were used (Table 2).

**TABLE 2:** Summary of groin hernia repairs and type of meshes used.

Operation and mesh type	Number of studies (%)
Lichtenstein repair	37
lightweight	19 (51)
heavyweight	13 (35)
mediumweight	0 (0)
light- and heavyweight	5 (14)
Laparoscopic repair	12
lightweight	2 (17)
heavyweight	8 (67)
mediumweight	0 (0)
light- and heavyweight	1 (8)
medium- and heavyweight	1 (8)

### Lightweight Mesh

**FIGURE 2:**
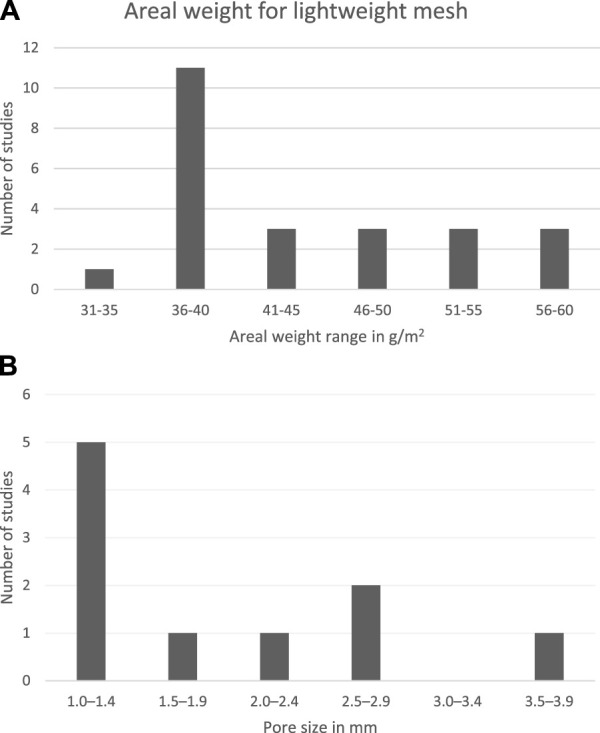
Definition of lightweight meshes regarding **(A)** areal weight and **(B)** pore size diameter in mm. For studies that provided a range, the mean of this range was calculated and used in the figure. Some areal weight and pore sizes were reported in an unspecified manner, and these were categorised in the range closest to the minimum estimate (i.e., >1 was classified in the range closest to 1 but also greater than 1).

A total of 26 lightweight meshes were reported in 25 studies (9–28, 50–55) (Table 1). The areal weight was reported in all but one study (21), with a median of 39 g/m^2^, an IQR of 36–50 g/m^2^, and a range of 35–60 g/m^2^ (Figure 2A). The only study that did not report the weight in g/m^2^ described the weight as “approximately 60% lighter weight than traditional polypropylene mesh” (21). The pore size was reported in 13 studies (9–11, 14, 18, 20, 22, 23, 28, 50–53) (Figure 2B). Two studies described that the lightweight mesh had large pore size without specifying the size in mm (10, 22), while the remaining ten studies either reported the pore diameter in mm or in µm, which was converted to mm (one study informed the size by email (23)) (9, 11, 14, 18, 20, 23, 50–53). Two studies had unspecified pore sizes (9, 53) and two studies used a range (14, 28). Nevertheless, the median of all lightweight meshes was 1.6 mm with an IQR of 1.0–2.3 mm and a range of 1.0–4.0 mm.

The mesh size was reported in cm in 15 studies (9, 13, 14, 17, 19, 20, 21, 23, 25, 26, 28, 51–54). There were many variations, which are presented in Table 1.

### Heavyweight Mesh

**FIGURE 3:**
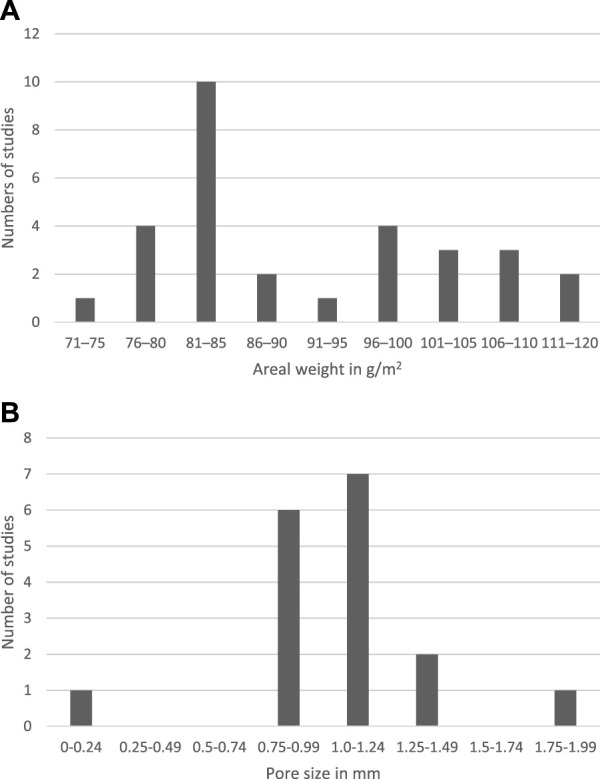
Definition of heavyweight meshes regarding areal weight **(A)**. Pore size for heavyweight meshes in mm **(B)**. For studies that provided a range, the mean of this range was calculated and used in the figure. Both for areal weight and for pore sizes reported in greater-than x were categorised in the closest ranging group.

A total of 30 heavyweight meshes were reported in 28 studies (29–56) (Table 1). For all heavyweight meshes, the areal weight was reported in g/m^2^ with a median areal weight of 88 g/m^2^, an IQR of 81–104 g/m^2^, and a range of 72–116 g/m^2^ (Figure 3A).

The pore size was reported in mm for 15 studies (33, 34, 36, 38–41, 45, 48, 49, 51–54, 56), with a median of 1.0 mm, an IQR of 0.84–1.0 mm, and a range of 0.08–1.8 mm (Figure 3B). One study that reported the mesh having 0.8–1.2 mm pores also described the mesh as having small pores (33), while another study only mentioned that the mesh was microporous without specifying the size in mm (28).

The mesh size was reported in 19 studies for 20 heavyweight meshes (29, 30, 33–35, 38, 40–44, 46, 47, 48, 51–54, 56). Nine of the meshes measured 10 × 15 cm (30, 33, 35, 42, 43, 47, 48, 56), but there were many variations (Table 1).

## Discussion

In this systematic scoping review, we reported how mesh weight was defined in randomised controlled trials on groin hernia repair. There seemed to be a distinct definition in the areal weight, where lightweight had an areal weight of ≤60 g/m^2^ and heavyweight had an areal weight of >70 g/m^2^. Pore sizes overlapped between lightweight and heavyweight meshes.

This study has several strengths. It is reported according to the PRISMA-ScR (6), and the protocol was registered in a public database before data extraction to increase transparency (7). We conducted a broad search using various databases with assistance from an information specialist, and two authors screened the titles and abstracts and the full text studies. Our study also has limitations. Only one author extracted data, but all data were reviewed for accuracy. Another limitation is that we only included English language studies. However, only including English language rarely compromises the review quality (68). Thirdly, ten studies could not be retrieved. Lastly, since this study’s main focus was on mesh weight and pore sizes it does not include other technical aspects of mesh properties such as elasticity, tensile strength, and other design properties of the mesh.

We need a universal classification based on the specific properties of the mesh as proposed by an international guideline on inguinal hernia management (1). However, this guideline (1) also points out that a universal classification is hard to achieve. In this study, we have investigated how RCTs have defined light–, medium–, and heavyweight mesh terms for flat polypropylene or polyester meshes. Only one study used a mediumweight mesh, and the nomenclature should therefore probably only comprise lightweight and heavyweight mesh. Even though there was some consensus regarding the areal mesh weight in g/m^2^, there was no general agreement of what small pores and large pores are and if lightweight and heavyweight meshes have characteristic pore sizes. Earlier studies have tried to categorise mesh weight classes. A study from 2008 proposed a classification as follows (69): ultralight weight <35 g/m^2^, lightweight 35–50 g/m^2^, mediumweight 51–90 g/m^2^, and heavyweight >90 g/m^2^. Another proposed classification from 2012 (70) emphasised that in the previous classification (69), a heavyweight mesh weighing 91 g/m^2^ would be in the same category as a heavyweight mesh weighing almost three times the weight. Thus, they proposed a classification that doubles the next limit: ultra-light <35 g/m^2^, light ≥35 <70 g/m^2^, standard ≥70 < 140 g/m^2^, and heavy ≥140 g/m^2^. Recently, meta-analyses comparing light- and heavyweight meshes in patients undergoing laparoscopic repair (5) or Lichtenstein repair (4) for uncomplicated inguinal hernia have defined lightweight meshes as ≤ 50 g/m^2^ and heavyweight meshes as >70 g/m^2^. Some of the lightweight meshes in this scoping review were over 50 g/m^2^, but the heavyweight meshes were in the same category as the proposed definition by the meta-analyses (4, 5). This underlines the problem with the classifications as mesh types fall under different categories. It is important to achieve a common technical language so that surgeons with different backgrounds and educational systems agree upon and utilise a common language. This would ease comparison in meta-analyses, thereby guiding clinical practice. However, the mesh market is in constant development, and with the current data presented here, we propose a simplified definition where lightweight could be all meshes with an areal weight ≤60 g/m^2^ and heavyweight meshes would be all meshes with an areal weight >70 g/m^2^.

In conclusion, the areal weight for lightweight and heavyweight meshes had a wide range, but all studies have defined lightweight as being ≤60 g/m^2^ and heavyweight as being >70 g/m^2^. There was an overlap between light- and heavyweight meshes’ pore sizes with a tendency that lightweight meshes had larger pore sizes.

## Footnotes

1 Mendeley (2022). Available from: https://www.mendeley.com (Accessed November 2, 2022).

2 Covidence (2022). Available from: https://www.covidence.org (Accessed November 2, 2022).
